# Acute cardiovascular hospitalizations and illness severity before and during the COVID‐19 pandemic

**DOI:** 10.1002/clc.23590

**Published:** 2021-03-07

**Authors:** Vishal N. Rao, Michelle D. Kelsey, Anita M. Kelsey, Stuart D. Russell, Robert J. Mentz, Manesh R. Patel, Marat Fudim

**Affiliations:** ^1^ Division of Cardiology Duke University Medical Center Durham North Carolina USA; ^2^ Duke Clinical Research Institute Duke University Medical Center Durham North Carolina USA

**Keywords:** cardiovascular disease, COVID‐19, heart failure, hospitalizations, mortality, risk scores

## Abstract

**Background:**

Cardiovascular disease (CVD) hospitalizations declined worldwide during the COVID‐19 pandemic. It is unclear how shelter‐in‐place orders affected acute CVD hospitalizations, illness severity, and outcomes.

**Hypothesis:**

COVID‐19 pandemic was associated with reduced acute CVD hospitalizations (heart failure [HF], acute coronary syndrome [ACS], and stroke [CVA]), and worse HF illness severity.

**Methods:**

We compared acute CVD hospitalizations at Duke University Health System before and after North Carolina's shelter‐in‐place order (January 1–March 29 vs. March 30–August 31), and used parallel comparison cohorts from 2019. We explored illness severity among admitted HF patients using ADHERE (“high risk”: >2 points) and GWTG‐HF (“>10%”: >57 points) in‐hospital mortality risk scores, as well as echocardiography‐derived parameters.

**Results:**

Comparing hospitalizations during January 1–March 29 (N = 1618) vs. March 30–August 31 (*N* = 2501) in 2020, mean daily CVD hospitalizations decreased (18.2 vs. 16.1 per day, *p* = .0036), with decreased length of stay (8.4 vs. 7.5 days, *p* = .0081) and no change in in‐hospital mortality (4.7 vs. 5.3%, *p* = .41). HF hospitalizations decreased (9.0 vs. 7.7 per day, *p* = .0019), with higher ADHERE (“high risk”: 2.5 vs. 4.5%; *p* = .030), but unchanged GWTG‐HF (“>10%”: 5.3 vs. 4.6%; *p* = .45), risk groups. Mean LVEF was lower (39.0 vs. 37.2%, *p* = .034), with higher mean LV mass (262.4 vs. 276.6 g, *p* = .014).

**Conclusions:**

CVD hospitalizations, HF illness severity, and echocardiography measures did not change between admission periods in 2019. Evaluating short‐term data, the COVID‐19 shelter‐in‐place order was associated with reductions in acute CVD hospitalizations, particularly HF, with no significant increase in in‐hospital mortality and only minor differences in HF illness severity.

**Abbreviations**
ACS: acute coronary syndrome
ADHERE: “Acute Decompensated Heart Failure National Registry”
COVID‐19: coronarvirus 2019
CVA: acute stroke syndrome
CVD: cardiovascular disease
DUHS: Duke University Health System
GWTG‐HF: “Get with The Guidelines®‐Heart Failure”
HF: heart failure
LOS: length of stay
LV: left ventricular
LVEF: left ventricular ejection fraction
NCDHHS: North Carolina Department of Health and Human Services
SARS‐CoV‐2: Severe Acute Respiratory Syndrome Coronavirus‐2
TTE: transthoracic echocardiography

## 1 INTRODUCTION

The United States has surpassed other nations to become the epicenter of the Severe Acute Respiratory Syndrome Coronavirus‐2 (SARS‐CoV‐2) pandemic, with recent numbers of confirmed coronavirus 2019 (COVID‐19) cases passing 86.6 million and 1.87 million COVID‐19‐related deaths in the world as of January 5, 2021.^1^ Hospitals across the US and Europe have reported a significant impact on cardiovascular disease (CVD)‐related hospitalizations due to the COVID‐19 pandemic.^2^, ^3^, ^4^, ^5^, ^6^, ^7^, ^8^, ^9^ With shelter‐in‐place orders issued to curb the spread of SARS‐CoV‐2, these mandates have additionally affected the numbers of total hospital admissions^8^ and raised concern for delayed access to emergent and urgent healthcare and excess CVD mortality.^2^ Acute CVD conditions, including acute heart failure (HF), acute chest coronary syndromes (ACS), and acute stroke syndromes (CVA) contribute to a large disease burden and account for the vast majority of cardiovascular‐related hospitalizations and deaths in the United States.^10^ There is growing concern that patients with prior CVD or CVD risk factors who develop new or worsening CVD conditions may choose to present late or not at all, positing significant risk of worsened morbidity and mortality due to delays in care. In light of new waves of COVID‐19 cases and considerations for new shelter‐in‐place orders across the US and worldwide, we sought to analyze the impact of the COVID‐19 pandemic and statewide shelter‐in‐place orders on acute CVD hospitalizations metrics and HF hospitalization illness severity measures before and after the first wave of COVID‐19 cases in North Carolina (NC).

## 2 METHODS

We conducted a retrospective study using data extracted from the Duke University Health System (DUHS) electronic health record and the Duke Echocardiography Laboratory Database. DUHS consists of three hospitals, with one academic tertiary care medical center, and two community‐based facilities, and serves a broad North Carolina population as well as a large referral‐based population. Incident COVID‐19 cases were extracted from the North Carolina Department of Health and Human Services (NCDHHS) COVID‐19 Data Dashboard.^11^ The study received approval from the Duke University Institutional Review Board.

This study included adult patients who were admitted to a DUHS hospital from January 1, 2019 to August 31, 2019 and January 1, 2020 to August 31, 2020 with a primary International Classification of Disease (ICD)‐9 or − 10 admission diagnosis of an acute CVD condition, including acute HF, ACS, or CVA (Supplemental Table 1). Patients less than 18 years of age at time of admission were not included. Patients who had scheduled outpatient visits within the hospital that did not result in an inpatient admission were also not included. Acute CVD conditions presenting concurrently at time of admission, while uncommon, were categorized as follows: (1) primary HF hospitalizations included HF only, both HF and ACS, or both HF and CVA; (2) primary ACS hospitalizations included ACS only or both ACS and CVA; (3) primary CVA hospitalizations included CVA only. Hospitalized patients were categorized by admission date into four comparison groups. Within 2020, we divided admission periods into before or after the NC executive shelter‐in‐place order on March 30, 2020,^12^ and analogous groups were divided into admission periods before or after March 30, 2019 for descriptive comparisons only. Final admissions periods were as follows: January 1–March 29, 2019, March 30–August 31, 2019, January 1–March 29, 2020, and March 30–August 31, 2020.

Patient characteristics collected from each hospitalization included demographics, vital signs, anthropometrics, and laboratory studies as recorded as the first available occurrence during hospitalizations, either in the Emergency Department or at the time of direct ward admission. Chronic obstructive pulmonary disease, a comorbid condition to cardiovascular disease, was extracted using prior historical or admission ICD‐9/−10 codes (Supplemental Table 1). Place of care was defined as the primary service that cared for the patient prior to hospital discharge. Discharge disposition was determined based on discharge documentation, and categorized as home (including with and without home‐based health services), facility (acute or skilled nursing care), or any hospice‐based care. Length of stay (LOS) was calculated from the date and time of admission to the date and time of discharge, to the nearest tenth decimal in days. Total and in‐hospital mortality were recorded for all hospitalizations.

HF‐specific mortality risk categories were derived from HF admission vital signs and laboratory data using the “Acute Decompensated Heart Failure National Registry” (ADHERE) and the “Get with The Guidelines®‐Heart Failure” (GWTG‐HF) algorithms.^13^, ^14^, ^15^ An ADHERE score of >2 was considered “high risk” and correlated with a 19.8–21.9% in‐hospital mortality risk from acute heart failure,^13^, ^15^ and GWTG‐HF score of >57 correlated with >10% in‐hospital all‐cause mortality risk.^14^ Transthoracic echocardiography (TTE) parameters were obtained for those admitted with acute HF either during the admission encounter, or, if not available, within 1.5 years prior to admission. TTE parameters included left ventricular ejection fraction (LVEF) and left ventricular (LV) mass using the linear method equation.^16^ We analyzed LVEF and LV mass since they are both prognostic clinical measures of hospitalized HF patients.^17^, ^18^, ^19^

We summarized baseline characteristics using descriptive statistics (mean for continuous variables, frequencies and percentages for binary and class variables). We compared hospitalization characteristics between admission periods and tested for differences of each measure using two‐sample Student's t‐test or one‐way analysis of variance (ANOVA) for continuous variables, and Pearson's Chi‐square or Fisher's Exact test for categorical variables. We performed post‐hoc pairwise comparisons between admission groups for significant differences across time periods. An exploratory analysis of HF risk characteristics was performed comparing 2019 to 2020 admission periods of March 2–March 29 (after NC first COVID‐19 case), and separately March 30–August 31 (after NC shelter‐in‐place order). For variables with few missing data (<5%), we imputed continuous variables to the overall median value, dichotomous variables to 'no,' and multichotomous variables to the most frequent categorical value. For variables with high missing data (>5%), we treated missing data as a separate category. We used a pre‐specified alpha of 0.05 to establish statistical significance and reported 95% confidence intervals. We also performed a sensitivity analysis among all primary CVD hospitalizations with a positive SARS‐CoV‐2 test to determine whether there was a differential effect on results by comorbid COVID‐19 diagnosis. All statistical analyses were performed using Stata 16 (StataCorp LP, College Station, Texas).

## 3 RESULTS

During January 1 to August 31 in 2019, there were a total of 4783 hospitalizations across DUHS for acute CVD conditions. Of these total hospitalizations, 2393 were primarily for HF (including 61 HF and ACS, 13 HF and CVA), 1111 were primarily for ACS (including 9 ACS and CVA), and 1279 were primarily for CVA. During January 1 to August 31 in 2020, there were a total of 4119 hospitalizations to the DUHS for acute CVD conditions. Of these total hospitalizations, 1991 were primarily for HF (including 51 HF and ACS, 16 HF and CVA), 956 were primarily for ACS (including 5 ACS and CVA), and 1172 were primarily for CVA.

Among the total 8902 hospitalizations, the mean age was 66.3 years, 44.4% were women, and 41.7% were African Americans. Thirty‐seven admitted patients were positive for SARS‐CoV‐2 (15 HF, 7 ACS, and 15 CVA). Baseline characteristics were generally similar across groups (Table 1). BMI differed across admission periods (F = 2.63, *p* = .048). We found no differences in the proportions of HF, ACS, and CVA hospitalization types across admission periods. We likewise found no difference in place of care. When compared with those admitted between January 1 and March 29, 2020, patients admitted after the NC shelter‐in‐place order had increased rates of home discharges. Similar differences were observed when comparing groups by admission year only (Supplemental Table 2). Exploratory analyses demonstrated reduction in daily total CVD, HF, and ACS admissions following the first NC COVID‐19 case in March 2, 2020 with 2019 as reference groups (Supplemental Table 3).

**TABLE 1:** Characteristics of acute cardiovascular disease admissions across the Duke Health System before and after the North Carolina stay‐at‐home order with 2019 as reference groups

Patient characteristics^a^	Jan 1 – Mar 29, 2019; *N* = 1747	March 30 – Aug 31, 2019; *N* = 3036	Jan 1 – Mar 29, 2020; *N* = 1618	Mar 30 – Aug 31, 2020; *N* = 2501	*p*
Age (yrs)	66.9 (14.5)	65.9 (15.3)	66.8 (14.5)	66.2 (14.4)	.095
Female	802 (45.9)	1322 (43.5)	737 (45.6)	1089 (43.5)	.25
Race					
Caucasian	931 (53.3)	1568 (51.6)	867 (53.6)	1299 (51.9)	.42
African American	717 (41.0)	1310 (43.2)	649 (40.1)	1040 (41.6)	
Asian	19 (1.1)	25 (0.8)	19 (1.2)	32 (1.3)	
Other	80 (4.6)	133 (4.4)	83 (5.1)	130 (5.2)	
Clinical characteristics					
Height (cm)	170.5 (10.8)	170.6 (10.9)	170.7 (10.7)	170.7 (11.3)	.92
Weight (kg)	88.4 (26.2)	89.2 (27.4)	89.4 (26.5)	90.0 (26.3)	.24
BMI (kg/m^2^)	30.5 (8.2)	30.8 (8.7)	30.8 (8.5)	31.3 (11.7)	.048
Systolic BP (mmHg)	130.3 (26.5)	130.8 (26.6)	130.9 (27.0)	129.5 (27.0)	.19
Diastolic BP (mmHg)	72.6 (17.2)	72.5 (17.4)	72.7 (17.1)	73.4 (17.7)	.27
Heart rate (bpm)	81.9 (17.5)	81.3 (16.9)	81.7 (17.2)	81.6 (16.8)	.72
Sodium (mEq/L)	136.9 (3.9)	137.3 (4.2)	136.9 (3.6)	137.3 (4.1)	.0001
BUN (mg/dl)	27.2 (21.8)	26.9 (20.6)	26.6 (21.1)	26.8 (20.9)	.86
Creatinine (mg/dl)	1.80 (1.95)	1.87 (2.10)	1.83 (1.94)	1.84 (2.11)	.70
Atrial fibrillation	632 (36.2)	1120 (36.9)	599 (37.0)	918 (36.7)	.96
COPD	372 (21.3)	717 (23.6)	372 (23.0)	530 (21.2)	.10
Primary hospitalization type					
HF	851 (48.7)	1468 (48.4)	778 (48.1)	1146 (45.8)	.41
HF & ACS	22 (1.3)	39 (1.3)	20 (1.2)	31 (1.2)	
HF & CVA	6 (0.3)	7 (0.2)	7 (0.4)	9 (0.4)	
ACS	392 (22.4)	710 (23.4)	371 (22.9)	580 (23.2)	
ACS & CVA	6 (0.3)	3 (0.1)	0 (0)	5 (0.2)	
CVA	470 (26.9)	809 (26.7)	442 (27.3)	730 (29.2)	
Places of care					
Cardiology	504 (28.8)	901 (29.7)	488 (30.2)	710 (28.4)	.77
Medicine	721 (41.3)	1243 (40.9)	655 (40.5)	1043 (41.7)	
Neurology	197 (11.3)	299 (9.8)	169 (10.4)	249 (10.0)	
Other	325 (18.6)	593 (19.5)	306 (18.9)	499 (19.9)	
Discharge location^b^					
Home	1248 (71.4)	2191 (72.2)	1146 (70.9)	1872 (76.2)	<.001
Facility	358 (20.5)	641 (21.1)	338 (20.9)	385 (15.7)	
Hospice	45 (2.6)	65 (2.1)	56 (3.5)	67 (2.7)	

Abbreviations: ACS, acute coronary syndromes; COPD, chronic obstructive pulmonary disease; CVA, cerebrovascular accidents; CVD, cardiovascular disease; HF, heart failure.

*Note*: There were a total of 8902 distinct admissions. Comparison groups were divided in 2019 and 2020 either before or after March 30 since the North Carolina Stay‐At‐Home order went into effect on March 30, 2020.

^a^ Values are mean ± SD for continuous variables or *n* (%) for categorical variables.

^b^ As of the time of this analysis (September 5, 2020), 47 patients remained hospitalized across all admission groups. Discharge location values do not reflect currently admitted patients or in‐hospital mortality.

There was a significant decrease in average daily acute CVD admissions across admission periods in 2019 and 2020 (F = 4.15, *p* = .0064). Comparing admission periods January 1–March 29 versus March 30–August 31 in 2020, daily CVD hospitalizations decreased, largely due to a decrease in daily HF, but not daily ACS or daily CVA hospitalizations (Table 2). When comparing the March 30–August 31 admission periods in 2019 versus 2020, there was a similar observed decline in daily CVD hospitalizations, largely due to both daily HF and daily ACS, but not daily CVA (Table 2). Across all four admission periods in 2019 and 2020, LOS did not change in admissions for ACS or CVA, but significantly decreased during HF hospitalizations (F = 3.91, *p* = .0084). Among those admitted for all primary CVD across the four admission periods in 2019 and 2020, there was no difference in the rate of in‐hospital mortality (χ^2^=2.94, *p* = .40; Table 2).

**TABLE 2:** Comparison of Acute Cardiovascular Disease Admissions across Duke Health System Before and After North Carolina Stay‐At‐Home Order with 2019 as Reference Groups

Patient characteristics^a^	Jan 1 – Mar 29, 2019	Mar 30 – Aug 31, 2019	Jan 1 – Mar 29, 2020	Mar 30 – Aug 31, 2020	*p*
All acute CVD	1747	3036	1618	2501	
Daily admission, mean	19.9 (5.1)	19.6 (5.0)	18.2 (5.2)	16.1 (5.2)	<.0001
Length of stay, days	8.5 (12.1)	7.9 (10.1)	8.4 (11.6)	7.5 (8.1)	.010
In‐hospital mortality, *N*	96 (5.5)	138 (4.6)	76 (4.7)	132 (5.3)	.40
Heart failure	879	1514	805	1186	
Daily admission, mean	10.0 (3.4)	9.8 (3.3)	9.0 (3.6)	7.7 (3.2)	<.0001
Length of stay, days	9.5 (14.3)	8.4 (11.1)	8.8 (10.9)	7.9 (7.8)	.0084
In‐hospital mortality, *N*	30 (3.4)	47 (3.1)	26 (3.2)	44 (3.7)	.85
ACS	398	713	371	585	
Daily admission, mean	4.5 (2.4)	4.6 (2.2)	4.2 (2.3)	3.8 (2.0)	.0064
Length of stay, days	6.2 (6.9)	6.3 (6.9)	6.3 (9.8)	5.6 (6.2)	.31
In‐hospital mortality, *N*	18 (4.5)	35 (4.9)	18 (4.8)	26 (4.4)	.98
CVA	470	809	442	730	
Daily admission, mean	5.3 (2.6)	5.2 (2.5)	5.0 (2.1)	4.7 (2.4)	.16
Length of stay, days	8.5 (10.6)	8.4 (10.5)	9.3 (13.8)	8.5 (9.7)	.58
In‐hospital mortality, *N*	48 (10.2)	56 (6.9)	32 (7.2)	62 (8.5)	.18

*Note*: There were a total of 8902 distinct admissions. The HF admissions category included HF only, HF & ACS (112), and HF & CVA (29). The ACS admissions category included ACS only and ACS & CVA (14). At the time of the analysis (September 5, 2020), a total of 47 patients remained hospitalized and were excluded from length of stay and in‐hospital mortality calculations (26 HF, 7 ACS, 14 CVA).

Abbreviations: CVD, cardiovascular disease; HF, heart failure; ACS, acute coronary syndromes; CVA, cerebrovascular accidents.

^a^ Values are mean ± SD for continuous variables or *n* (%) for categorical variables.

Acute HF hospitalization illness severity and transthoracic echocardiography measures are reported in Table 3. We found an increase in the proportion of high and intermediate risk patients admitted for acute HF between January 1–March 29 and March 30–August 31 admission periods in 2020 based on ADHERE risk, but no difference in ADHERE risk categories across all four HF admission periods in 2019 and 2020. There was a difference in GWTG‐HF mortality risk categories across all four admissions groups in 2019 and 2020, which was largely due to increased rates of higher risk HF admissions when comparing March 30–August 31 admission periods in 2019 vs. 2020. Exploratory analyses comparing 2019 to 2020 admission periods between March 2–March 29, and separately March 30–August 31, demonstrated worse ADHERE and GWTG‐HF risk scores after the NC shelter‐in‐place order, but not after first NC COVID‐19 case (Supplemental Table 4). Among the total 4384 HF admissions, there were 2907 (66%) available TTEs with LVEF and 2782 (63%) with LV mass during or prior to each distinct HF hospitalization. We found small, but statistically significant decreases in LVEF (39.0 vs. 37.2%; *p* = .034) and increases in LV mass (262.4 vs. 276.6 g, *p* = .014) between January 1–March 29 and March 30–August 31 in 2020, which were not present among the 2019 admission groups (Figure 2). Despite N‐terminal pro‐brain natriuretic peptide levels available for 2016 (46%) of all HF admissions, there were no differences in natriuretic peptide levels across four admission periods (Table 3).

**TABLE 3:** acute heart failure admission characteristics and illness severity across the Duke Health System Before and after the North Carolina stay‐at‐home order with 2019 as reference groups

HF admission groups, N		Jan 1 – Mar 29, 2019 (879)	Mar 30 – Aug 31, 2019 (1514)	*p* ^a^	Jan 1 – Mar 29, 2020 (805)	Mar 30 – Aug 31, 2020 (1186)	*p* ^a^	*p* ^b^
TTE^c^	LVEF %, mean (SD)	37.9 (15.0)	38.5 (15.2)	.44	39.0 (15.1)	37.2 (15.4)	.034	.14
	LV Mass in g grams, mean (SD)	269.0 (101.2)	273.2 (104.2)	.45	262.4 (95.1)	276.6 (104.9)	.014	.090
ADHERE	Mortality risk group, *N* (%)							
	Low	437 (49.7)	779 (51.4)	.56	431 (53.5)	588 (49.6)	.030	.052
	Intermediate	415 (47.2)	697 (46.0)		354 (44.0)	545 (45.9)		
	High	27 (3.1)	38 (2.5)		20 (2.5)	53 (4.5)		
GWTG‐HF	Mortality risk group, *N* (%)							
	<1%	174 (19.8)	324 (21.4)	.38	189 (23.5)	248 (20.9)	.45	.046
	>1–5%	588 (66.9)	1022 (67.5)		495 (61.5)	762 (64.2)		
	>5–10%	83 (9.4)	117 (7.7)		78 (9.7)	121 (10.2)		
	>10%	34 (3.9)	51 (3.4)		43 (5.3)	55 (4.6)		
Labs	NT‐proBNP (pg/ml), mean (SD)^d^	14 387 (30040)	13 524 (28691)	.93	13 451 (23906)	13 266 (27137)	1.0	.94

Abbreviations: HF, heart failure; LOS, length of stay; TTE, transthoracic echocardiography; LVEF, left ventricular ejection fraction; LV, left ventricular; ADHERE, “Acute Decompensated Heart Failure National Registry Algorithm”; GWTG‐HF, Get with The Guidelines® ‐ Heart Failure Risk Score; NT‐proBNP, N‐terminal pro‐brain natriuretic peptide.

*Note*: The HF admissions category included HF only, HF & ACS (112), and HF & CVA (29). Groups within 2019 and 2020 were divided into before or after March 30 since the North Carolina Stay at Home order went into effect on March 30, 2020.

^a^ *p*‐value compares difference between heart failure admission groups in January 1 – March 29 and March 30 – August 31 of the same year.

^b^ Terminal p‐value compares difference across all four groups in 2019 and 2020.

^c^ There were 2907 available TTE studies of the 4384 total HF admissions. Comparisons for LVEF are of 2907 TTEs (562 between January 1 and March 29, 2019; 1017 between March 30 and August 31, 2019; 532 between January 1 and March 29, 2020; 796 between March 30 and August 31, 2020). Comparisons for LV mass are of 2782 TTEs (540 between January 1 and March 29, 2019; 964 between March 30 and August 31, 2019; 507 between January 1 and March 29, 2020; 771 between March 30 and August 31, 2020).

^d^ NT‐proBNP was available in only 46% of all HF admissions.

**FIGURE 1:**
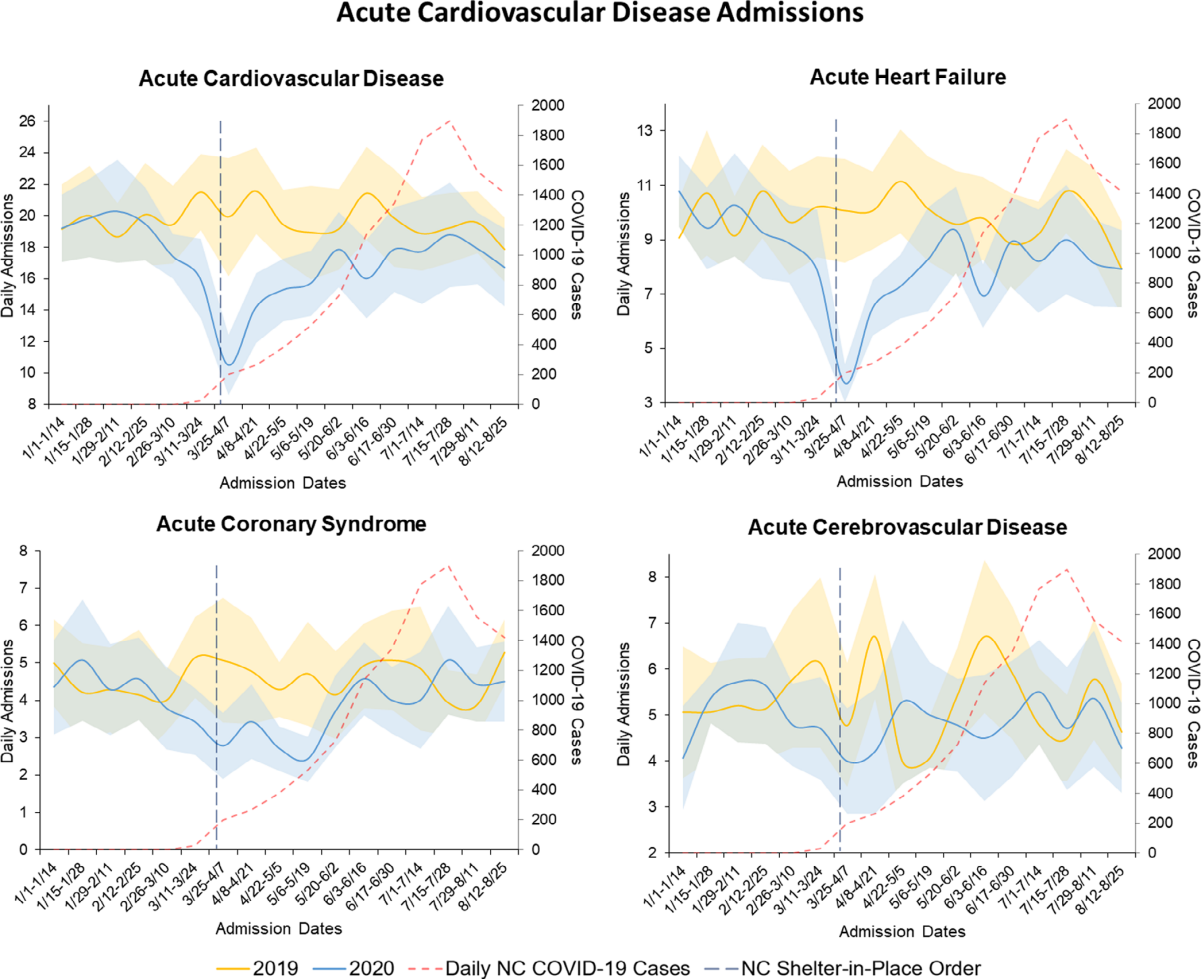
(Central Illustration) Trends in Daily acute cardiovascular disease admission across Duke University Health System from January to August in 2019 and 2020 and North Carolina COVID‐19 cases. Trends in average daily admissions and 95% confidence intervals for acute cardiovascular disease conditions, including acute heart failure, acute coronary syndromes, and acute cerebrovascular accidents, across the Duke University Health System and average North Carolina daily reported new COVID‐19 cases

According to the NCDHHS COVID‐19 Dashboard, the first reported NC COVID‐19 case was on March 2, 2020.^11^ Average daily NC COVID‐19 cases peaked to 1900 new cases per day between July 15 and July 28, 2020 (Figure 1, Central Illustration). Sensitivity analyses excluding the 37 patients positive for SARS‐CoV‐2/COVID‐19 from all aforementioned analyses did not change any of the associations observed across time periods and by admission type.

## 4 DISCUSSION

We present a timely analysis describing the impact of the COVID‐19 pandemic and a statewide shelter‐in‐place order on hospitalization metrics for acute CVD, including HF, ACS, and CVA, with a detailed description of risk profiles of admitted patients with HF. There was a significant reduction in average daily CVD admissions across a large academic health system after the NC shelter‐in‐place order on March 30, 2020. When evaluated separately, this reduction was driven primarily by a decrease in HF hospitalizations, with a significant, though less substantial, decrease in ACS hospitalizations. Despite a common concern that patients hospitalized after shelter‐in‐place orders may be sicker due to delayed care, our HF cohort had only a small change in validated in‐hospital mortality risk scores and no change in in‐hospital mortality. Admission rates in our study rebounded to 2019 levels within 10 weeks from the first reported COVID‐19 case in North Carolina on March 2, 2020^11^ and within 6 weeks from the shelter‐in‐place order on March 30, 2020 (Figure 1, Central Illustration).

The results of CVD admission trends in our study are consistent with data that has been reported at other institutions at the outset of the COVID‐19 pandemic.^2^, ^3^, ^4^, ^5^, ^6^, ^7^, ^8^, ^9^ Bhatt et al. found a decrease in daily CVD hospitalizations in March 2020 (−5.9% hospitalizations per day, 95% CI: −7.6% to −4.3%; *p* < .001) across a tertiary care health system in New England.^2^ Bhatt et al. described similar reductions across HF, ACS, and CVA categories; whereas, we observed that the most prominent change in average daily admissions were among HF hospitalizations (Table 2). Other centers, in Mississippi, United States,^6^ Tennessee, United States,^4^ London, United Kingdom,^3^ noted significant reductions in HF hospitalizations when examined independently.

Only one other study has characterized changes in HF hospitalizations during the COVID‐19 pandemic after shelter‐in‐place orders were lifted. Ling et al. described a return to prior baseline hospitalization rates within 2 weeks of lifting the shelter‐in‐place orders in Georgia, United States.^8^ In the present study, we found HF admission rates returned to 2019 levels within 6 weeks after implementation of the NC shelter‐in‐place order, and within 2 weeks prior to lifting this order on May 23, 2020.^12^ Notably, Ling et al. noted no evidence of a “hospitalization debt” with no significant increase in hospitalizations after shelter‐in‐place orders were lifted. This observed phenomenon is consistent with our findings as well.

There was concern at the outset of the COVID‐19 pandemic that patients presenting to the hospital would represent a sicker population, due to delayed access to care and enhanced COVID‐19 infection risk with underlying heart failure.^20^, ^21^ Using the validated prognostic risk scores and echocardiography parameters, we found only a small yet significant difference in illness severity before and after March 30, 2020. The GWTG‐HF risk scores were not different between the same 2020 admission periods, but there were higher proportions of “High Risk” HF admissions after March 30 when comparing 2019 versus 2020 admission periods. The ADHERE risk scores were likewise similar between groups. Although these two risk models use different numbers of clinical variables, they have demonstrated relatively similar predictive performance,^13^, ^22^ consistent with our observations. There was a statistically significant difference in LVEF from before and after the NC shelter‐in‐place order in 2020 (37.2% vs. 39.0%; *p* = .034), yet we suspect that this degree of change has minimal clinical significance. Differences in HF risk characteristics were similar in our exploratory analyses comparing 2019 to 2020 after the NC shelter‐in‐place order on March 30, but not after the first NC COVID‐19 case on March 2. We additionally found no difference in measured in‐hospital HF mortality across 2019 and 2020 admission periods. Despite the small observed differences across multiple risk prediction tools, admitted HF patients were clinically similar in health before and after March 30 in 2020. While other studies have described worsened peripheral edema and higher proportions of New York Heart Association class III or IV symptoms among hospitalized HF patients during the COVID‐19 pandemic,^3^ these physical exam findings alone may not directly correlate with in‐hospital mortality risk. Our study is the first to provide insight on HF hospitalization illness severity using validated risk scores and imaging instruments during the COVID‐19 pandemic.

There are many possible reasons why HF hospitalizations decreased during the study period. Successful telemedicine implementation potentially maintained outpatient care for HF patients.^21^ We observed an increase in telehealth visits for CVD after the NC shelter‐in‐place order (data not shown), which was consistent with prior published analyses on outpatient CVD care at our own institution.^23^ Providers may also have managed worsening HF symptoms in same‐day access clinics or observation units to prevent hospital admissions.^24^, ^25^ Additionally, patients may have been more willing to increase oral diuretics than in the past, or they may have had fewer acute exacerbations resulting from improved dietary influences on fluid retention due to restaurant closures. Alternatively, newly‐developed or acute on chronic HF that otherwise would have been hospitalized during March and April 2020 may have resulted in out‐of‐hospital deaths or presented to other regional hospital systems. Further research incorporating forthcoming statewide mortality data may better define the interplay between behavioral factors and access to healthcare during this time.

**FIGURE 2:**
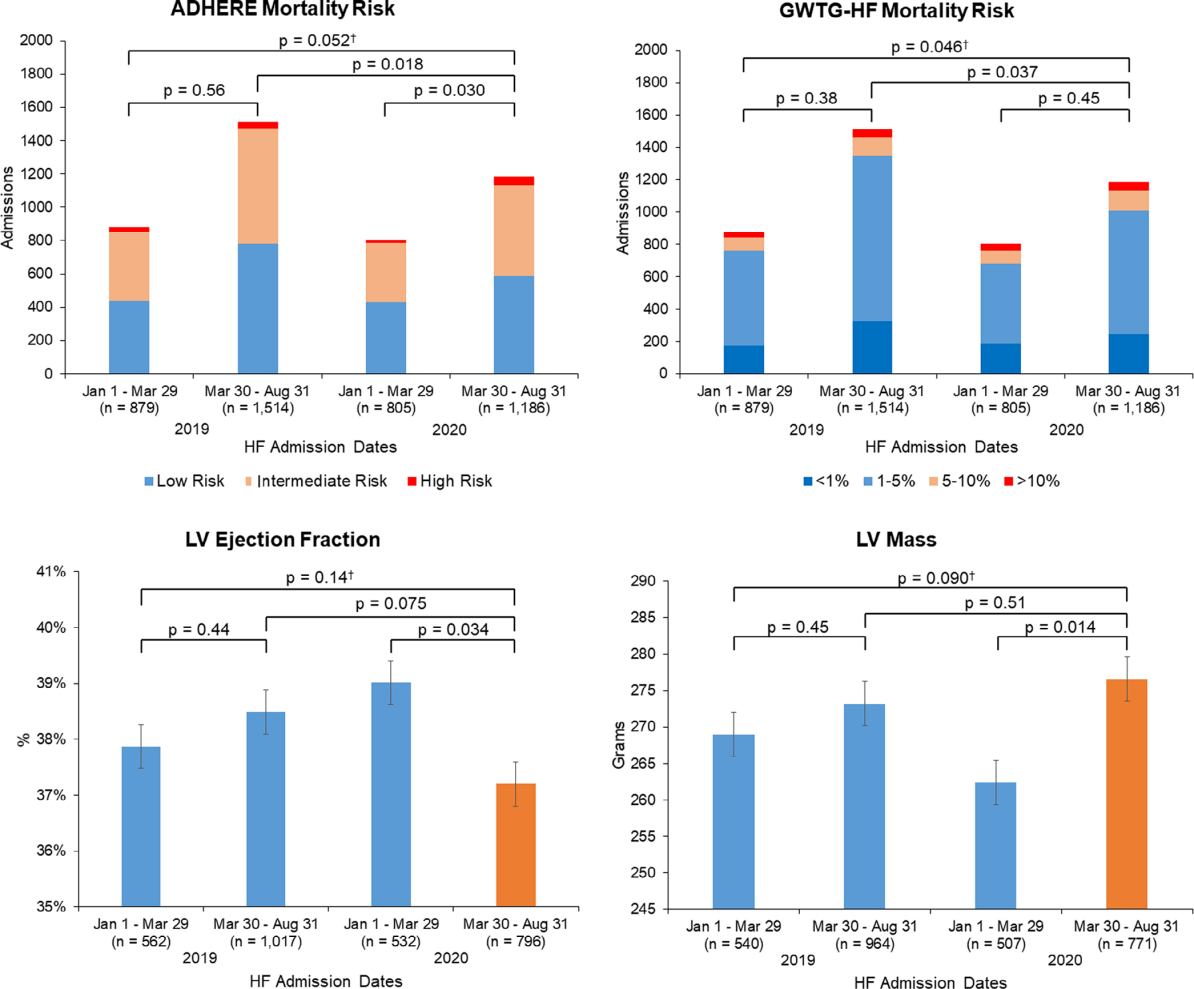
Illness severity markers for heart failure admissions before and after the North Carolina stay‐at‐home order with 2019 as reference groups. Heart failure (HF) illness severity estimated by calculated HF in‐hospital mortality risk including the Acute Decompensated Heart Failure National Registry Algorithm (ADHERE) and Get with The Guidelines®‐Heart Failure (GWTG‐HF) risk scores, as well as available echocardiography‐derived left ventricular (LV) ejection fraction and LV mass. Comparison of ADHERE risk scores, LV ejection fraction, and LV mass demonstrated a significant but only slightly sicker group of HF patients admitted after the NC Stay at Home Order in 2020. GWTG‐HF risk was slightly worse only when comparing March 30 to August 31 admission periods in 2019 versus 2020. (†p refers to comparison of estimates across all four groups)

We identify several limitations in the present study. First, this was a retrospective, descriptive analysis and was unable to systematically evaluate the effect of covariates on admission metrics and illness severity markers during the study periods. Second, our results cannot directly evaluate for causation between any of the factors associated with the COVID‐19 pandemic and our observations among admission characteristics. Third, among the illness severity markers, there were only 63–66% available TTEs for all HF admissions, which limited our ability to describe LVEF and LV mass for the whole cohort. Since HF is a clinical diagnosis based on physical exam, laboratory values, and other imaging modalities, not all patients hospitalized for HF had TTE within the specified timeframe, and referred patients may have had TTE outside of the DUHS. Further, we occasionally relied on clinically available TTE data, from up to 1.5 years before hospitalization, which may not accurately reflect the current condition. Fourth, we used administrative ICD‐9/10 coding based on clinical‐driven primary CVD diagnoses which may not include all CVD patients. While patients improperly coded or misclassified may not have been captured, we used the same diagnostic criteria across all study periods, minimizing the impact of that limitation. Finally, illness with COVID‐19 that had resulted in acute CVD but was not coded for acute CVD were not captured (Figure 2).

Despite these caveats, the data we have presented may inform local policies on shelter‐in‐place orders. These restrictions, coupled with increasing rates of COVID‐19, are associated with changes in acute cardiovascular admissions. Though we do not know what impact this may have had on overall mortality, these changes should be considered as lawmakers and healthcare officials weigh strategies to combat the COVID‐19 pandemic and future case surges.

## 5 CONCLUSION

Acute CVD admissions, including HF, ACS, and CVA, decreased after the onset of COVID‐19 and the NC shelter‐in‐place order in large academic health system. Despite the reduction in HF admissions, there was only a small difference in mortality risk measured by ADHERE, but not GWTG‐HF, risk scores among those admitted, and there was no change in in‐hospital mortality across CVD or HF admission groups during the study period. Future studies may better define the interplay between the COVID‐19 pandemic, shelter‐in‐place orders, and hospitalization trends for CVD conditions and CVD illness severity across health systems in the US.

## CONFLICT OF INTEREST

Vishal N. Rao, Michelle D. Kelsey, and Stuart D. Russell have no disclosures. Marat Fudim consults for AstraZeneca, AxonTherapies, CVRx, Daxor, Edwards LifeSciences, Galvani, NXT Biomedical, Respicardia. Anita M. Kelsey is a consultant for Lantheus Medical Imaging. Robert J. Mentz received research support and honoraria from Abbott, American Regent, Amgen, AstraZeneca, Bayer, Boehringer Ingelheim/Eli Lilly, Boston Scientific, Cytokinetics, Fast BioMedical, Gilead, Innolife, Medtronic, Merck, Novartis, Relypsa, Respicardia, Roche, Sanofi, Vifor, and Windtree Therapeutics. MRP receives research grants from AstraZeneca, Bayer, Janssen, Medtronic, NHLBI, Heartflow, and Phillips, and serves on the advisory board for Bayer, Janssen, and Heartflow.

## Supporting information


**Supplemental Table 1** Primary Admission ICD‐9/−10 Codes
**Supplemental Table 2**: Characteristics of Acute Cardiovascular Disease Admissions across Duke Health System between January to August in 2019 and 2020*
**Supplemental Table 3**: Exploratory Analyses of Acute Cardiovascular Disease Admissions across Duke Health System from March 2 to August 31 Separated by the North Carolina Stay‐At‐Home Order with 2019 as Reference Groups*
**Supplemental Table 4**: Exploratory Analyses of Acute Heart Failure Admission Characteristics and Illness Severity across the Duke Health System from March 2 to August 31 Separated by the North Carolina Stay‐At‐Home Order with 2019 as Reference Groups*
