# Supplementary material for: Acute cardiovascular hospitalizations and illness severity before and during the COVID‐19 pandemic
Source: Clin Cardiol. 2021 Mar 7;44(5):656–64. doi: 10.1002/clc.23590 (PMC8119829; doi:10.1002/clc.23590)
Supplement: Supplementary file 1 — Supplemental Table 1 Primary Admission ICD‐9/−10 Codes Supplemental Table 2: Characteristics of Acute Cardiovascular Disease Admissions across Duke Health System between January to August in 2019 and 2020* Supplemental Table 3: Exploratory Analyses of Acute Cardiovascular Disease Admissions across Duke Health System from March 2 to August 31 Separated by the North Carolina Stay‐At‐Home Order with 2019 as Reference Groups* Supplemental Table 4: Exploratory Analyses of Acute Heart Failure Admission Characteristics and Illness Severity across the Duke Health System from March 2 to August 31 Separated by the North Carolina Stay‐At‐Home Order with 2019 as Reference Groups* [file CLC-44--s001.docx]

**Supplemental Table 1: Primary Admission ICD-9/-10 Codes**

| Heart Failure ICD Codes | I50.41 | Acute combined systolic (congestive) and diastolic (congestive) heart failure | I42.0 | Dilated cardiomyopathy |
| --- | --- | --- | --- | --- |
|  | 428.41 | Acute combined systolic and diastolic heart failure | I50.84 | End stage heart failure |
|  | I50.31 | Acute diastolic (congestive) heart failure | I42.4 | Endocardial fibroelastosis |
|  | 428.31 | Acute diastolic heart failure | I42.3 | Endomyocardial (eosinophilic) disease |
|  | I50.43 | Acute on chronic combined systolic (congestive) and diastolic (congestive) heart failure | I50 | Heart failure |
|  | 428.43 | Acute on chronic combined systolic and diastolic heart failure | I50.9 | Heart failure, unspecified |
|  | I50.33 | Acute on chronic diastolic (congestive) heart failure | T86.22 | Heart transplant failure |
|  | 428.33 | Acute on chronic diastolic heart failure | I50.83 | High output heart failure |
|  | I50.813 | Acute on chronic right heart failure | I13.0 | Hypertensive heart and chronic kidney disease with heart failure and stage 1 through stage 4 chronic kidney disease, or unspecified chronic kidney disease |
|  | I50.23 | Acute on chronic systolic (congestive) heart failure | I13.2 | Hypertensive heart and chronic kidney disease with heart failure and with stage 5 chronic kidney disease, or end stage renal disease |
|  | 428.23 | Acute on chronic systolic heart failure | 404.13 | Hypertensive heart and chronic kidney disease, benign, with heart failure and chronic kidney disease stage V or end stage renal disease |
|  | I50.811 | Acute right heart failure | 404.11 | Hypertensive heart and chronic kidney disease, benign, with heart failure and with chronic kidney disease stage I through stage IV, or unspecified |
|  | I50.21 | Acute systolic (congestive) heart failure | 404.01 | Hypertensive heart and chronic kidney disease, malignant, with heart failure and with chronic kidney disease stage I through stage IV, or unspecified |
|  | 428.21 | Acute systolic heart failure | 404.03 | Hypertensive heart and chronic kidney disease, malignant, with heart failure and with chronic kidney disease stage V or end stage renal disease |
|  | I42.6 | Alcoholic cardiomyopathy | 404.93 | Hypertensive heart and chronic kidney disease, unspecified, with heart failure and chronic kidney disease stage V or end stage renal disease |
|  | 402.11 | Benign hypertensive heart disease with heart failure | 404.91 | Hypertensive heart and chronic kidney disease, unspecified, with heart failure and with chronic kidney disease stage I through stage IV, or unspecified |
|  | I50.82 | Biventricular heart failure | I11.0 | Hypertensive heart disease with heart failure |
|  | I42.7 | Cardiomyopathy due to drug and external agent | 425.11 | Hypertrophic obstructive cardiomyopathy |
|  | I43 | Cardiomyopathy in diseases classified elsewhere | I25.5 | Ischemic cardiomyopathy |
|  | I42.9 | Cardiomyopathy, unspecified | I50.1 | Left ventricular failure, unspecified |
|  | I50.42 | Chronic combined systolic (congestive) and diastolic (congestive) heart failure | 402.01 | Malignant hypertensive heart disease with heart failure |
|  | 428.42 | Chronic combined systolic and diastolic heart failure | I51.4 | Myocarditis, unspecified |
|  | I50.32 | Chronic diastolic (congestive) heart failure | I42.1 | Obstructive hypertrophic cardiomyopathy |
|  | 428.32 | Chronic diastolic heart failure | I42.8 | Other cardiomyopathies |
|  | I50.812 | Chronic right heart failure | I50.89 | Other heart failure |
|  | I50.22 | Chronic systolic (congestive) heart failure | I42.2 | Other hypertrophic cardiomyopathy |
|  | 428.22 | Chronic systolic heart failure | I09.9 | Rheumatic heart disease, unspecified |
|  | 428.4 | Combined systolic and diastolic heart failure | I09.81 | Rheumatic heart failure |
|  | 428 | Congestive heart failure, unspecified | 398.91 | Rheumatic heart failure (congestive) |
|  | I50.3 | Diastolic (congestive) heart failure | I50.814 | Right heart failure due to left heart failure |
|  | 428.3 | Diastolic heart failure | I50.810 | Right heart failure, unspecified |
|  | I51.89 | Other ill-defined heart diseases | 425.9 | Secondary cardiomyopathy, unspecified |
|  | I42.5 | Other restrictive cardiomyopathy | 428.2 | Systolic heart failure |
|  | O90.3 | Peripartum cardiomyopathy | I51.81 | Takotsubo syndrome |
|  | 674.53 | Peripartum cardiomyopathy, antepartum condition or complication | I50.40 | Unspecified combined systolic (congestive) and diastolic (congestive) heart failure |
|  | 674.51 | Peripartum cardiomyopathy, delivered, with or without mention of antepartum condition | I50.30 | Unspecified diastolic (congestive) heart failure |
|  | 674.54 | Peripartum cardiomyopathy, postpartum condition or complication | 402.91 | Unspecified hypertensive heart disease with heart failure |
|  | I97.130 | Postprocedural heart failure following cardiac surgery | I50.20 | Unspecified systolic (congestive) heart failure |
|  | I97.131 | Postprocedural heart failure following other surgery | Z95.811 | Presence of heart assist device |
|  | Z95.812 | Presence of fully implantable artificial heart |  |  |
| Acute Coronary Syndrome ICD codes | 411.81 | Acute coronary occlusion without myocardial infarction | 429.79 | Certain sequelae of myocardial infarction, not elsewhere classified, other |
|  | I24.0 | Acute coronary thrombosis not resulting in myocardial infarction | I25.42 | Coronary artery dissection |
|  | I24.9 | Acute ischemic heart disease, unspecified | 414.12 | Dissection of coronary artery |
|  | 410 | Acute myocardial infarction of anterolateral wall, episode of care unspecified | I24.1 | Dressler's syndrome |
|  | 410.01 | Acute myocardial infarction of anterolateral wall, initial episode of care | 411.1 | Intermediate coronary syndrome |
|  | 410.02 | Acute myocardial infarction of anterolateral wall, subsequent episode of care | I21.4 | Non-ST elevation (NSTEMI) myocardial infarction |
|  | 410.2 | Acute myocardial infarction of inferolateral wall, episode of care unspecified | 411.89 | Other acute and subacute forms of ischemic heart disease, other |
|  | 410.21 | Acute myocardial infarction of inferolateral wall, initial episode of care | 413.9 | Other and unspecified angina pectoris |
|  | 410.22 | Acute myocardial infarction of inferolateral wall, subsequent episode of care | I23.8 | Other current complications following acute myocardial infarction |
|  | 410.3 | Acute myocardial infarction of inferoposterior wall, episode of care unspecified | I24.8 | Other forms of acute ischemic heart disease |
|  | 410.31 | Acute myocardial infarction of inferoposterior wall, initial episode of care | I20.8 | Other forms of angina pectoris |
|  | 410.32 | Acute myocardial infarction of inferoposterior wall, subsequent episode of care | I23.7 | Postinfarction angina |
|  | 410.1 | Acute myocardial infarction of other anterior wall, episode of care unspecified | 413.1 | Prinzmetal angina |
|  | 410.11 | Acute myocardial infarction of other anterior wall, initial episode of care | I23.3 | Rupture of cardiac wall without hemopericardium as current complication following acute myocardial infarction |
|  | 410.12 | Acute myocardial infarction of other anterior wall, subsequent episode of care | I23.5 | Rupture of papillary muscle as current complication following acute myocardial infarction |
|  | 410.4 | Acute myocardial infarction of other inferior wall, episode of care unspecified | I25.6 | Silent myocardial ischemia |
|  | 410.41 | Acute myocardial infarction of other inferior wall, initial episode of care | I21.02 | ST elevation (STEMI) myocardial infarction involving left anterior descending coronary artery |
|  | 410.42 | Acute myocardial infarction of other inferior wall, subsequent episode of care | I21.21 | ST elevation (STEMI) myocardial infarction involving left circumflex coronary artery |
|  | 410.5 | Acute myocardial infarction of other lateral wall, episode of care unspecified | I21.01 | ST elevation (STEMI) myocardial infarction involving left main coronary artery |
|  | 410.51 | Acute myocardial infarction of other lateral wall, initial episode of care | I21.09 | ST elevation (STEMI) myocardial infarction involving other coronary artery of anterior wall |
|  | 410.52 | Acute myocardial infarction of other lateral wall, subsequent episode of care | I21.19 | ST elevation (STEMI) myocardial infarction involving other coronary artery of inferior wall |
|  | 410.8 | Acute myocardial infarction of other specified sites, episode of care unspecified | I21.29 | ST elevation (STEMI) myocardial infarction involving other sites |
|  | 410.81 | Acute myocardial infarction of other specified sites, initial episode of care | I21.11 | ST elevation (STEMI) myocardial infarction involving right coronary artery |
|  | 410.82 | Acute myocardial infarction of other specified sites, subsequent episode of care | I21.3 | ST elevation (STEMI) myocardial infarction of unspecified site |
|  | 410.9 | Acute myocardial infarction of unspecified site | T82.855A | Stenosis of coronary artery stent, initial encounter |
|  | 410.9 | Acute myocardial infarction of unspecified site, episode of care unspecified | T82.855S | Stenosis of coronary artery stent, sequela |
|  | 410.91 | Acute myocardial infarction of unspecified site, initial episode of care | T82.855D | Stenosis of coronary artery stent, subsequent encounter |
|  | 410.92 | Acute myocardial infarction of unspecified site, subsequent episode of care | 410.7 | Subendocardial infarction, episode of care unspecified |
|  | I21.9 | Acute myocardial infarction, unspecified | 410.71 | Subendocardial infarction, initial episode of care |
|  | I20 | Angina pectoris | 410.72 | Subendocardial infarction, subsequent episode of care |
|  | I20.1 | Angina pectoris with documented spasm | I22.2 | Subsequent non-ST elevation (NSTEMI) myocardial infarction |
|  | I20.9 | Angina pectoris, unspecified | I22.0 | Subsequent ST elevation (STEMI) myocardial infarction of anterior wall |
|  | I25.721 | Atherosclerosis of autologous artery coronary artery bypass graft(s) with angina pectoris with documented spasm | I22.1 | Subsequent ST elevation (STEMI) myocardial infarction of inferior wall |
|  | I25.728 | Atherosclerosis of autologous artery coronary artery bypass graft(s) with other forms of angina pectoris | I22.8 | Subsequent ST elevation (STEMI) myocardial infarction of other sites |
|  | I25.729 | Atherosclerosis of autologous artery coronary artery bypass graft(s) with unspecified angina pectoris | I22.9 | Subsequent ST elevation (STEMI) myocardial infarction of unspecified site |
|  | I25.720 | Atherosclerosis of autologous artery coronary artery bypass graft(s) with unstable angina pectoris | I23.6 | Thrombosis of atrium, auricular appendage, and ventricle as current complications following acute myocardial infarction |
|  | I25.711 | Atherosclerosis of autologous vein coronary artery bypass graft(s) with angina pectoris with documented spasm | 410.6 | True posterior wall infarction, episode of care unspecified |
|  | I25.718 | Atherosclerosis of autologous vein coronary artery bypass graft(s) with other forms of angina pectoris | 410.61 | True posterior wall infarction, initial episode of care |
|  | I25.719 | Atherosclerosis of autologous vein coronary artery bypass graft(s) with unspecified angina pectoris | 410.62 | True posterior wall infarction, subsequent episode of care |
|  | I25.710 | Atherosclerosis of autologous vein coronary artery bypass graft(s) with unstable angina pectoris | I20.0 | Unstable angina |
|  | I25.761 | Atherosclerosis of bypass graft of coronary artery of transplanted heart with angina pectoris with documented spasm | I23.2 | Ventricular septal defect as current complication following acute myocardial infarction |
|  | I25.768 | Atherosclerosis of bypass graft of coronary artery of transplanted heart with other forms of angina pectoris | I25.739 | Atherosclerosis of nonautologous biological coronary artery bypass graft(s) with unspecified angina pectoris |
|  | I25.769 | Atherosclerosis of bypass graft of coronary artery of transplanted heart with unspecified angina pectoris | I25.791 | Atherosclerosis of other coronary artery bypass graft(s) with angina pectoris with documented spasm |
|  | I25.760 | Atherosclerosis of bypass graft of coronary artery of transplanted heart with unstable angina | I25.798 | Atherosclerosis of other coronary artery bypass graft(s) with other forms of angina pectoris |
|  | I25.701 | Atherosclerosis of coronary artery bypass graft(s), unspecified, with angina pectoris with documented spasm | I25.799 | Atherosclerosis of other coronary artery bypass graft(s) with unspecified angina pectoris |
|  | I25.708 | Atherosclerosis of coronary artery bypass graft(s), unspecified, with other forms of angina pectoris | I25.790 | Atherosclerosis of other coronary artery bypass graft(s) with unstable angina pectoris |
|  | I25.709 | Atherosclerosis of coronary artery bypass graft(s), unspecified, with unspecified angina pectoris | I25.111 | Atherosclerotic heart disease of native coronary artery with angina pectoris with documented spasm |
|  | I25.700 | Atherosclerosis of coronary artery bypass graft(s), unspecified, with unstable angina pectoris | I25.118 | Atherosclerotic heart disease of native coronary artery with other forms of angina pectoris |
|  | I25.751 | Atherosclerosis of native coronary artery of transplanted heart with angina pectoris with documented spasm | I25.119 | Atherosclerotic heart disease of native coronary artery with unspecified angina pectoris |
|  | I25.758 | Atherosclerosis of native coronary artery of transplanted heart with other forms of angina pectoris | I25.110 | Atherosclerotic heart disease of native coronary artery with unstable angina pectoris |
|  | I25.759 | Atherosclerosis of native coronary artery of transplanted heart with unspecified angina pectoris | I23.1 | Atrial septal defect as current complication following acute myocardial infarction |
|  | I25.750 | Atherosclerosis of native coronary artery of transplanted heart with unstable angina | I25.738 | Atherosclerosis of nonautologous biological coronary artery bypass graft(s) with other forms of angina pectoris |
|  | I25.731 | Atherosclerosis of nonautologous biological coronary artery bypass graft(s) with angina pectoris with documented spasm | I25.730 | Atherosclerosis of nonautologous biological coronary artery bypass graft(s) with unstable angina pectoris |
| Cerebrovascular Accident ICD codes | I69.320 | Aphasia following cerebral infarction | 435.8 | Other specified transient cerebral ischemias |
|  | I69.120 | Aphasia following nontraumatic intracerebral hemorrhage | I69.328 | Other speech and language deficits following cerebral infarction |
|  | I69.020 | Aphasia following nontraumatic subarachnoid hemorrhage | I69.128 | Other speech and language deficits following nontraumatic intracerebral hemorrhage |
|  | I69.220 | Aphasia following other nontraumatic intracranial hemorrhage | I69.028 | Other speech and language deficits following nontraumatic subarachnoid hemorrhage |
|  | I69.390 | Apraxia following cerebral infarction | I69.228 | Other speech and language deficits following other nontraumatic intracranial hemorrhage |
|  | I69.190 | Apraxia following nontraumatic intracerebral hemorrhage | I69.318 | Other symptoms and signs involving cognitive functions following cerebral infarction |
|  | I69.090 | Apraxia following nontraumatic subarachnoid hemorrhage | I69.118 | Other symptoms and signs involving cognitive functions following nontraumatic intracerebral hemorrhage |
|  | I69.290 | Apraxia following other nontraumatic intracranial hemorrhage | I69.018 | Other symptoms and signs involving cognitive functions following nontraumatic subarachnoid hemorrhage |
|  | I69.393 | Ataxia following cerebral infarction | I69.218 | Other symptoms and signs involving cognitive functions following other nontraumatic intracranial hemorrhage |
|  | I69.193 | Ataxia following nontraumatic intracerebral hemorrhage | G45.8 | Other transient cerebral ischemic attacks and related syndromes |
|  | I69.093 | Ataxia following nontraumatic subarachnoid hemorrhage | 346.6 | Persistent migraine aura with cerebral infarction |
|  | I69.293 | Ataxia following other nontraumatic intracranial hemorrhage | G43.611 | Persistent migraine aura with cerebral infarction, intractable, with status migrainosus |
|  | I69.310 | Attention and concentration deficit following cerebral infarction | G43.619 | Persistent migraine aura with cerebral infarction, intractable, without status migrainosus |
|  | I69.110 | Attention and concentration deficit following nontraumatic intracerebral hemorrhage | G43.609 | Persistent migraine aura with cerebral infarction, not intractable, without status migrainosus |
|  | I69.010 | Attention and concentration deficit following nontraumatic subarachnoid hemorrhage | 346.61 | Persistent migraine aura with cerebral infarction, with intractable migraine, so stated, without mention of status migrainosus |
|  | I69.210 | Attention and concentration deficit following other nontraumatic intracranial hemorrhage | V12.54 | Personal history of transient ischemic attack (TIA), and cerebral infarction without residual deficits |
|  | G46.3 | Brain stem stroke syndrome | I69.313 | Psychomotor deficit following cerebral infarction |
|  | G46.4 | Cerebellar stroke syndrome | 430 | Subarachnoid hemorrhage |
|  | 434.9 | Cerebral artery occlusion unspecified | G45.9 | Transient cerebral ischemic attack, unspecified |
|  | 434.91 | Cerebral artery occlusion, unspecified with cerebral infarction | 432.9 | Unspecified intracranial hemorrhage |
|  | 434.11 | Cerebral embolism with cerebral infarction | I69.30 | Unspecified sequelae of cerebral infarction |
|  | I63.6 | Cerebral infarction due to cerebral venous thrombosis, nonpyogenic | I69.10 | Unspecified sequelae of nontraumatic intracerebral hemorrhage |
|  | I63.12 | Cerebral infarction due to embolism of basilar artery | I69.00 | Unspecified sequelae of nontraumatic subarachnoid hemorrhage |
|  | I63.423 | Cerebral infarction due to embolism of bilateral anterior cerebral arteries | I69.20 | Unspecified sequelae of other nontraumatic intracranial hemorrhage |
|  | I63.133 | Cerebral infarction due to embolism of bilateral carotid arteries | I69.319 | Unspecified symptoms and signs involving cognitive functions following cerebral infarction |
|  | I63.443 | Cerebral infarction due to embolism of bilateral cerebellar arteries | I69.119 | Unspecified symptoms and signs involving cognitive functions following nontraumatic intracerebral hemorrhage |
|  | I63.413 | Cerebral infarction due to embolism of bilateral middle cerebral arteries | I69.019 | Unspecified symptoms and signs involving cognitive functions following nontraumatic subarachnoid hemorrhage |
|  | I63.433 | Cerebral infarction due to embolism of bilateral posterior cerebral arteries | I69.219 | Unspecified symptoms and signs involving cognitive functions following other nontraumatic intracranial hemorrhage |
|  | I63.113 | Cerebral infarction due to embolism of bilateral vertebral arteries | 435.9 | Unspecified transient cerebral ischemia |
|  | I63.422 | Cerebral infarction due to embolism of left anterior cerebral artery | I69.312 | Visuospatial deficit and spatial neglect following cerebral infarction |
|  | I63.132 | Cerebral infarction due to embolism of left carotid artery | I69.112 | Visuospatial deficit and spatial neglect following nontraumatic intracerebral hemorrhage |
|  | I63.442 | Cerebral infarction due to embolism of left cerebellar artery | I69.012 | Visuospatial deficit and spatial neglect following nontraumatic subarachnoid hemorrhage |
|  | I63.412 | Cerebral infarction due to embolism of left middle cerebral artery | I69.212 | Visuospatial deficit and spatial neglect following other nontraumatic intracranial hemorrhage |
|  | I63.432 | Cerebral infarction due to embolism of left posterior cerebral artery | I60.21 | Nontraumatic subarachnoid hemorrhage from right anterior communicating artery |
|  | I63.112 | Cerebral infarction due to embolism of left vertebral artery | I60.01 | Nontraumatic subarachnoid hemorrhage from right carotid siphon and bifurcation |
|  | I63.49 | Cerebral infarction due to embolism of other cerebral artery | I60.11 | Nontraumatic subarachnoid hemorrhage from right middle cerebral artery |
|  | I63.19 | Cerebral infarction due to embolism of other precerebral artery | I60.31 | Nontraumatic subarachnoid hemorrhage from right posterior communicating artery |
|  | I63.421 | Cerebral infarction due to embolism of right anterior cerebral artery | I60.51 | Nontraumatic subarachnoid hemorrhage from right vertebral artery |
|  | I63.131 | Cerebral infarction due to embolism of right carotid artery | I60.00 | Nontraumatic subarachnoid hemorrhage from unspecified carotid siphon and bifurcation |
|  | I63.441 | Cerebral infarction due to embolism of right cerebellar artery | I60.7 | Nontraumatic subarachnoid hemorrhage from unspecified intracranial artery |
|  | I63.411 | Cerebral infarction due to embolism of right middle cerebral artery | I60.10 | Nontraumatic subarachnoid hemorrhage from unspecified middle cerebral artery |
|  | I63.431 | Cerebral infarction due to embolism of right posterior cerebral artery | I60.30 | Nontraumatic subarachnoid hemorrhage from unspecified posterior communicating artery |
|  | I63.111 | Cerebral infarction due to embolism of right vertebral artery | I60.9 | Nontraumatic subarachnoid hemorrhage, unspecified |
|  | I63.429 | Cerebral infarction due to embolism of unspecified anterior cerebral artery | 434 | Occlusion of cerebral arteries |
|  | I63.139 | Cerebral infarction due to embolism of unspecified carotid artery | I63.8 | Other cerebral infarction |
|  | I63.449 | Cerebral infarction due to embolism of unspecified cerebellar artery | I63.89 | Other cerebral infarction |
|  | I63.40 | Cerebral infarction due to embolism of unspecified cerebral artery | I63.81 | Other cerebral infarction due to occlusion or stenosis of small artery |
|  | I63.419 | Cerebral infarction due to embolism of unspecified middle cerebral artery | I67.848 | Other cerebrovascular vasospasm and vasoconstriction |
|  | I63.439 | Cerebral infarction due to embolism of unspecified posterior cerebral artery | I61.8 | Other nontraumatic intracerebral hemorrhage |
|  | I63.10 | Cerebral infarction due to embolism of unspecified precerebral artery | I60.8 | Other nontraumatic subarachnoid hemorrhage |
|  | I63.119 | Cerebral infarction due to embolism of unspecified vertebral artery | I69.364 | Other paralytic syndrome following cerebral infarction affecting left non-dominant side |
|  | I63.02 | Cerebral infarction due to thrombosis of basilar artery | I69.361 | Other paralytic syndrome following cerebral infarction affecting right dominant side |
|  | I63.323 | Cerebral infarction due to thrombosis of bilateral anterior cerebral arteries | I69.369 | Other paralytic syndrome following cerebral infarction affecting unspecified side |
|  | I63.033 | Cerebral infarction due to thrombosis of bilateral carotid arteries | I69.365 | Other paralytic syndrome following cerebral infarction, bilateral |
|  | I63.313 | Cerebral infarction due to thrombosis of bilateral middle cerebral arteries | I69.161 | Other paralytic syndrome following nontraumatic intracerebral hemorrhage affecting right dominant side |
|  | I63.333 | Cerebral infarction due to thrombosis of bilateral posterior cerebral arteries | I69.165 | Other paralytic syndrome following nontraumatic intracerebral hemorrhage, bilateral |
|  | I63.013 | Cerebral infarction due to thrombosis of bilateral vertebral arteries | I69.065 | Other paralytic syndrome following nontraumatic subarachnoid hemorrhage, bilateral |
|  | I63.322 | Cerebral infarction due to thrombosis of left anterior cerebral artery | I69.265 | Other paralytic syndrome following other nontraumatic intracranial hemorrhage, bilateral |
|  | I63.032 | Cerebral infarction due to thrombosis of left carotid artery | I69.098 | Other sequelae following nontraumatic subarachnoid hemorrhage |
|  | I63.342 | Cerebral infarction due to thrombosis of left cerebellar artery | I69.398 | Other sequelae of cerebral infarction |
|  | I63.312 | Cerebral infarction due to thrombosis of left middle cerebral artery | I69.198 | Other sequelae of nontraumatic intracerebral hemorrhage |
|  | I63.332 | Cerebral infarction due to thrombosis of left posterior cerebral artery | I69.298 | Other sequelae of other nontraumatic intracranial hemorrhage |
|  | I63.012 | Cerebral infarction due to thrombosis of left vertebral artery | I69.331 | Monoplegia of upper limb following cerebral infarction affecting right dominant side |
|  | I63.39 | Cerebral infarction due to thrombosis of other cerebral artery | I69.333 | Monoplegia of upper limb following cerebral infarction affecting right non-dominant side |
|  | I63.09 | Cerebral infarction due to thrombosis of other precerebral artery | I69.339 | Monoplegia of upper limb following cerebral infarction affecting unspecified side |
|  | I63.0 | Cerebral infarction due to thrombosis of precerebral arteries | I69.134 | Monoplegia of upper limb following nontraumatic intracerebral hemorrhage affecting left non-dominant side |
|  | I63.321 | Cerebral infarction due to thrombosis of right anterior cerebral artery | I69.131 | Monoplegia of upper limb following nontraumatic intracerebral hemorrhage affecting right dominant side |
|  | I63.031 | Cerebral infarction due to thrombosis of right carotid artery | I69.133 | Monoplegia of upper limb following nontraumatic intracerebral hemorrhage affecting right non-dominant side |
|  | I63.341 | Cerebral infarction due to thrombosis of right cerebellar artery | I69.139 | Monoplegia of upper limb following nontraumatic intracerebral hemorrhage affecting unspecified |
|  | I63.311 | Cerebral infarction due to thrombosis of right middle cerebral artery | I69.034 | Monoplegia of upper limb following nontraumatic subarachnoid hemorrhage affecting left non-dominant side |
|  | I63.331 | Cerebral infarction due to thrombosis of right posterior cerebral artery | I69.231 | Monoplegia of upper limb following other nontraumatic intracranial hemorrhage affecting right dominant side |
|  | I63.011 | Cerebral infarction due to thrombosis of right vertebral artery | I61.3 | Nontraumatic intracerebral hemorrhage in brain stem |
|  | I63.329 | Cerebral infarction due to thrombosis of unspecified anterior cerebral artery | I61.4 | Nontraumatic intracerebral hemorrhage in cerebellum |
|  | I63.039 | Cerebral infarction due to thrombosis of unspecified carotid artery | I61.1 | Nontraumatic intracerebral hemorrhage in hemisphere, cortical |
|  | I63.349 | Cerebral infarction due to thrombosis of unspecified cerebellar artery | I61.0 | Nontraumatic intracerebral hemorrhage in hemisphere, subcortical |
|  | I63.30 | Cerebral infarction due to thrombosis of unspecified cerebral artery | I61.2 | Nontraumatic intracerebral hemorrhage in hemisphere, unspecified |
|  | I63.319 | Cerebral infarction due to thrombosis of unspecified middle cerebral artery | I61.5 | Nontraumatic intracerebral hemorrhage, intraventricular |
|  | I63.339 | Cerebral infarction due to thrombosis of unspecified posterior cerebral artery | I61.6 | Nontraumatic intracerebral hemorrhage, multiple localized |
|  | I63.00 | Cerebral infarction due to thrombosis of unspecified precerebral artery | I61.9 | Nontraumatic intracerebral hemorrhage, unspecified |
|  | I63.019 | Cerebral infarction due to thrombosis of unspecified vertebral artery | I62.9 | Nontraumatic intracranial hemorrhage, unspecified |
|  | I63.22 | Cerebral infarction due to unspecified occlusion or stenosis of basilar artery | I60.2 | Nontraumatic subarachnoid hemorrhage from anterior communicating artery |
|  | I63.523 | Cerebral infarction due to unspecified occlusion or stenosis of bilateral anterior cerebral arteries | I60.4 | Nontraumatic subarachnoid hemorrhage from basilar artery |
|  | I63.233 | Cerebral infarction due to unspecified occlusion or stenosis of bilateral carotid arteries | I60.22 | Nontraumatic subarachnoid hemorrhage from left anterior communicating artery |
|  | I63.543 | Cerebral infarction due to unspecified occlusion or stenosis of bilateral cerebellar arteries | I60.02 | Nontraumatic subarachnoid hemorrhage from left carotid siphon and bifurcation |
|  | I63.513 | Cerebral infarction due to unspecified occlusion or stenosis of bilateral middle cerebral arteries | I60.12 | Nontraumatic subarachnoid hemorrhage from left middle cerebral artery |
|  | I63.533 | Cerebral infarction due to unspecified occlusion or stenosis of bilateral posterior cerebral arteries | I60.32 | Nontraumatic subarachnoid hemorrhage from left posterior communicating artery |
|  | I63.213 | Cerebral infarction due to unspecified occlusion or stenosis of bilateral vertebral arteries | I60.52 | Nontraumatic subarachnoid hemorrhage from left vertebral artery |
|  | I63.522 | Cerebral infarction due to unspecified occlusion or stenosis of left anterior cerebral artery | I69.154 | Hemiplegia and hemiparesis following nontraumatic intracerebral hemorrhage affecting left non-dominant side |
|  | I63.232 | Cerebral infarction due to unspecified occlusion or stenosis of left carotid arteries | I69.151 | Hemiplegia and hemiparesis following nontraumatic intracerebral hemorrhage affecting right dominant side |
|  | I63.542 | Cerebral infarction due to unspecified occlusion or stenosis of left cerebellar artery | I69.153 | Hemiplegia and hemiparesis following nontraumatic intracerebral hemorrhage affecting right non-dominant side |
|  | I63.512 | Cerebral infarction due to unspecified occlusion or stenosis of left middle cerebral artery | I69.159 | Hemiplegia and hemiparesis following nontraumatic intracerebral hemorrhage affecting unspecified side |
|  | I63.532 | Cerebral infarction due to unspecified occlusion or stenosis of left posterior cerebral artery | I69.054 | Hemiplegia and hemiparesis following nontraumatic subarachnoid hemorrhage affecting left non-dominant side |
|  | I63.212 | Cerebral infarction due to unspecified occlusion or stenosis of left vertebral artery | I69.051 | Hemiplegia and hemiparesis following nontraumatic subarachnoid hemorrhage affecting right dominant side |
|  | I63.59 | Cerebral infarction due to unspecified occlusion or stenosis of other cerebral artery | I69.059 | Hemiplegia and hemiparesis following nontraumatic subarachnoid hemorrhage affecting unspecified side |
|  | I63.29 | Cerebral infarction due to unspecified occlusion or stenosis of other precerebral arteries | I69.252 | Hemiplegia and hemiparesis following other nontraumatic intracranial hemorrhage affecting left dominant side |
|  | I63.521 | Cerebral infarction due to unspecified occlusion or stenosis of right anterior cerebral artery | I69.254 | Hemiplegia and hemiparesis following other nontraumatic intracranial hemorrhage affecting left non-dominant side |
|  | I63.231 | Cerebral infarction due to unspecified occlusion or stenosis of right carotid arteries | I69.251 | Hemiplegia and hemiparesis following other nontraumatic intracranial hemorrhage affecting right dominant side |
|  | I63.541 | Cerebral infarction due to unspecified occlusion or stenosis of right cerebellar artery | I69.253 | Hemiplegia and hemiparesis following other nontraumatic intracranial hemorrhage affecting right non-dominant side |
|  | I63.511 | Cerebral infarction due to unspecified occlusion or stenosis of right middle cerebral artery | I69.259 | Hemiplegia and hemiparesis following other nontraumatic intracranial hemorrhage affecting unspecified side |
|  | I63.531 | Cerebral infarction due to unspecified occlusion or stenosis of right posterior cerebral artery | 431 | Intracerebral hemorrhage |
|  | I63.211 | Cerebral infarction due to unspecified occlusion or stenosis of right vertebral artery | I69.311 | Memory deficit following cerebral infarction |
|  | I63.529 | Cerebral infarction due to unspecified occlusion or stenosis of unspecified anterior cerebral artery | I69.111 | Memory deficit following nontraumatic intracerebral hemorrhage |
|  | I63.239 | Cerebral infarction due to unspecified occlusion or stenosis of unspecified carotid artery | I69.011 | Memory deficit following nontraumatic subarachnoid hemorrhage |
|  | I63.549 | Cerebral infarction due to unspecified occlusion or stenosis of unspecified cerebellar artery | I69.211 | Memory deficit following other nontraumatic intracranial hemorrhage |
|  | I63.50 | Cerebral infarction due to unspecified occlusion or stenosis of unspecified cerebral artery | I69.342 | Monoplegia of lower limb following cerebral infarction affecting left dominant side |
|  | I63.519 | Cerebral infarction due to unspecified occlusion or stenosis of unspecified middle cerebral artery | I69.344 | Monoplegia of lower limb following cerebral infarction affecting left non-dominant side |
|  | I63.539 | Cerebral infarction due to unspecified occlusion or stenosis of unspecified posterior cerebral artery | I69.341 | Monoplegia of lower limb following cerebral infarction affecting right dominant side |
|  | I63.20 | Cerebral infarction due to unspecified occlusion or stenosis of unspecified precerebral arteries | I69.343 | Monoplegia of lower limb following cerebral infarction affecting right non-dominant side |
|  | I63.219 | Cerebral infarction due to unspecified occlusion or stenosis of unspecified vertebral artery | I69.349 | Monoplegia of lower limb following cerebral infarction affecting unspecified side |
|  | I63.9 | Cerebral infarction, unspecified | I69.144 | Monoplegia of lower limb following nontraumatic intracerebral hemorrhage affecting left non-dominant side |
|  | I67.82 | Cerebral ischemia | I69.141 | Monoplegia of lower limb following nontraumatic intracerebral hemorrhage affecting right dominant side |
|  | 434.01 | Cerebral thrombosis with cerebral infarction | I69.041 | Monoplegia of lower limb following nontraumatic subarachnoid hemorrhage affecting right dominant side |
|  | I69.31 | Cognitive deficits following cerebral infarction | I69.334 | Monoplegia of upper limb following cerebral infarction affecting left non-dominant side |
|  | I69.11 | Cognitive deficits following nontraumatic intracerebral hemorrhage | I69.114 | Frontal lobe and executive function deficit following nontraumatic intracerebral hemorrhage |
|  | I69.01 | Cognitive deficits following nontraumatic subarachnoid hemorrhage | I69.014 | Frontal lobe and executive function deficit following nontraumatic subarachnoid hemorrhage |
|  | I69.21 | Cognitive deficits following other nontraumatic intracranial hemorrhage | I69.214 | Frontal lobe and executive function deficit following other nontraumatic intracranial hemorrhage |
|  | I69.315 | Cognitive social or emotional deficit following cerebral infarction | I69.352 | Hemiplegia and hemiparesis following cerebral infarction affecting left dominant side |
|  | I69.322 | Dysarthria following cerebral infarction | I69.354 | Hemiplegia and hemiparesis following cerebral infarction affecting left non-dominant side |
|  | I69.122 | Dysarthria following nontraumatic intracerebral hemorrhage | I69.351 | Hemiplegia and hemiparesis following cerebral infarction affecting right dominant side |
|  | I69.022 | Dysarthria following nontraumatic subarachnoid hemorrhage | I69.353 | Hemiplegia and hemiparesis following cerebral infarction affecting right non-dominant side |
|  | I69.222 | Dysarthria following other nontraumatic intracranial hemorrhage | I69.359 | Hemiplegia and hemiparesis following cerebral infarction affecting unspecified side |
|  | I69.391 | Dysphagia following cerebral infarction | I69.152 | Hemiplegia and hemiparesis following nontraumatic intracerebral hemorrhage affecting left dominant side |
|  | I69.191 | Dysphagia following nontraumatic intracerebral hemorrhage | I69.092 | Facial weakness following nontraumatic subarachnoid hemorrhage |
|  | I69.091 | Dysphagia following nontraumatic subarachnoid hemorrhage | I69.292 | Facial weakness following other nontraumatic intracranial hemorrhage |
|  | I69.291 | Dysphagia following other nontraumatic intracranial hemorrhage | I69.323 | Fluency disorder following cerebral infarction |
|  | I69.321 | Dysphasia following cerebral infarction | I69.123 | Fluency disorder following nontraumatic intracerebral hemorrhage |
|  | I69.121 | Dysphasia following nontraumatic intracerebral hemorrhage | I69.223 | Fluency disorder following other nontraumatic intracranial hemorrhage |
|  | I69.021 | Dysphasia following nontraumatic subarachnoid hemorrhage | I69.314 | Frontal lobe and executive function deficit following cerebral infarction |
|  | I69.392 | Facial weakness following cerebral infarction | I69.192 | Facial weakness following nontraumatic intracerebral hemorrhage |
|  | I60.6 | Nontraumatic subarachnoid hemorrhage from other intracranial arteries |  |  |
| Chronic Obstructive Pulmonary Disease ICD Codes | J44.1 | Chronic obstructive pulmonary disease with (acute) exacerbation |  |  |
|  | J44.0 | Chronic obstructive pulmonary disease with (acute) lower respiratory infection |  |  |
|  | J44.9 | Chronic obstructive pulmonary disease, unspecified |  |  |
|  | 491.2 | Obstructive chronic bronchitis |  |  |

| **Supplemental Table 2: Characteristics of Acute Cardiovascular Disease Admissions across Duke Health System between January to August in 2019 and 2020*** | | | | |
| --- | --- | --- | --- | --- |
| **Patient Characteristics**^†^ | **Combined**  **(n=8,902)** | **Jan to Aug 2019**  **(n=4,783)** | **Jan to Aug 2020**  **(n=4,119)** | **p** |
| **Age (yrs)** | 66.3 (14.8) | 66.2 (15.0) | 66.4 (14.4) | 0.52 |
| **Female** | 3950 (44.4) | 2124 (44.4) | 1826 (44.3) | 0.94 |
| **Race** |  |  |  |  |
| **Caucasian** | 4664 (52.4) | 2499 (52.2) | 2166 (52.6) | 0.14 |
| **African American** | 3716 (41.7) | 2027 (42.4) | 1689 (41.0) |  |
| **Asian** | 95 (1.1) | 44 (0.9) | 51 (1.2) |  |
| **Other** | 426 (4.8) | 213 (4.4) | 213 (5.2) |  |
| **Clinical Characteristics** |  |  |  |  |
| **Height (cm)** | 170.6 (11.0) | 170.6 (10.8) | 170.7 (11.1) | 0.54 |
| **Weight (kg)** | 89.3 (26.7) | 88.9 (27.0) | 89.8 (26.4) | 0.11 |
| **BMI (kg/m^2^)** | 30.9 (9.5) | 30.7 (8.6) | 31.1 (10.6) | ***0.032*** |
| **Systolic BP (mmHg)** | 130.4 (26.8) | 130.6 (26.6) | 130.0 (27.0) | 0.30 |
| **Diastolic BP (mmHg)** | 72.8 (17.4) | 72.6 (17.3) | 73.1 (17.5) | 0.12 |
| **Heart Rate (bpm)** | 81.6 (17.0) | 81.5 (17.1) | 81.7 (17.0) | 0.67 |
| **Sodium (mEq/L)** | 137.1 (4.0) | 137.2 (4.1) | 137.1 (3.9) | 0.70 |
| **BUN (mg/dL)** | 26.9 (21.0) | 27.0 (21.1) | 26.7 (21.0) | 0.48 |
| **Creatinine (mg/dL)** | 1.84 (2.05) | 1.85 (2.04) | 1.83 (2.05) | 0.72 |
| **Atrial fibrillation** | 3269 (36.7) | 1752 (36.6) | 1517 (36.8) | 0.85 |
| **COPD** | 1991 (22.4) | 1089 (22.8) | 902 (21.9) | 0.33 |
| **Primary Hospitalization Type** |  |  |  |  |
| **HF**  **HF & ACS**  **HF & CVA** | 4243 (47.7)  112 (1.3)  29 (0.3) | 2319 (48.5)  61 (1.3)  13 (0.3) | 1924 (46.7)  51 (1.2)  16 (0.4) | 0.37 |
| **ACS**  **ACS & CVA** | 2053 (23.1)  14 (0.2) | 1102 (23.0)  9 (0.2) | 951 (23.1)  5 (0.1) |  |
| **Cerebrovascular Accident** | 2451 (27.5) | 1279 (26.7) | 1172 (28.4) |  |
| **Places of Care** |  |  |  |  |
| **Cardiology** | 2603 (29.2) | 1405 (29.4) | 1198 (29.1) | 0.96 |
| **Medicine** | 3662 (41.1) | 1964 (41.1) | 1,698 (41.2) |  |
| **Neurology** | 914 (10.3) | 496 (10.4) | 418 (10.2) |  |
| **Other** | 1723 (19.4) | 918  (19.2) | 805 (19.5) |  |
| **Discharge Location**^‡^ |  |  |  |  |
| **Home** | 6457 (72.9) | 3439 (71.9) | 3018 (74.1) | ***0.001*** |
| **Facility** | 1722 (19.4) | 999 (20.9) | 723 (17.8) |  |
| **Hospice** | 233 (2.63) | 110 (2.3) | 123 (3.0) |  |

*CVD, cardiovascular disease; HF, heart failure; ACS, acute coronary syndromes; CVA, cerebrovascular accidents; COPD, chronic obstructive pulmonary disease. There were a total of 8,902 distinct admissions.

†Values are mean ± SD for continuous variables or n (%) for categorical variables.

‡As of the time of this analysis, 47 patients remained hospitalized across all admission groups. Discharge location values do not reflect currently admitted patients or in-hospital mortality.

| **Supplemental Table 3: Exploratory Analyses of Acute Cardiovascular Disease Admissions across Duke Health System from March 2 to August 31 Separated by the North Carolina Stay-At-Home Order with 2019 as Reference Groups*** | | | | | | |
| --- | --- | --- | --- | --- | --- | --- |
| **Patient Characteristics†** | **Mar 2 – Mar 29, 2019** | **Mar 2 – Mar 29, 2020** | **p** | **Mar 30 – Aug 31, 2019** | **Mar 30 – Aug 31, 2020** | **p** |
| **All Acute CVD** | 569 | 423 |  | 3,036 | 2,501 |  |
| **Daily admission, mean** | 20.3 (4.9) | 15.1 (4.9) | ***0.001*** | 19.6 (5.0) | 16.1 (5.2) | ***<0.001*** |
| **Length of Stay, days** | 8.4 (9.9) | 7.9 (11.3) | 0.81 | 7.9 (10.1) | 7.5 (8.1) | 0.52 |
| **In-hospital Mortality, N** | 20 (3.5) | 18 (4.3) | 0.55 | 138 (4.6) | 132 (5.3) | 0.21 |
| **Heart Failure** | 285 | 203 |  | 1,514 | 1,186 |  |
| **Daily admission, mean** | 10.2 (3.7) | 7.2 (4.0) | ***0.006*** | 9.8 (3.3) | 7.7 (3.2) | ***<0.001*** |
| **Length of Stay, days** | 9.4 (10.9) | 7.8 (8.9) | 0.28 | 8.4 (11.1) | 7.9 (7.8) | 0.66 |
| **In-hospital Mortality, N** | 3 (1.0) | 6 (3.0) | 0.12 | 47 (3.1) | 44 (3.7) | 0.39 |
| **ACS** | 133 | 91 |  | 713 | 585 |  |
| **Daily admission, mean** | 4.7 (2.3) | 3.2 (1.9) | ***0.042*** | 4.6 (2.2) | 3.8 (2.0) | ***0.004*** |
| **Length of Stay, days** | 7.1 (8.8) | 5.4 (4.9) | 0.28 | 6.3 (6.9) | 5.6 (6.2) | 0.26 |
| **In-hospital Mortality, N** | 4 (3.0) | 5 (5.5) | 0.35 | 35 (4.9) | 26 (4.4) | 0.69 |
| **CVA** | 151 | 129 |  | 809 | 730 |  |
| **Daily admission, mean** | 5.4 (3.2) | 4.6 (2.1) | 0.64 | 5.2 (2.5) | 4.7 (2.4) | 0.28 |
| **Length of Stay, days** | 7.8 (8.7) | 9.7 (16.5) | 0.41 | 8.4 (10.5) | 8.5 (9.7) | 1.0 |
| **In-hospital Mortality, N** | 13 (8.6) | 7 (5.4) | 0.30 | 56 (6.9) | 62 (8.5) | 0.25 |

* CVD, cardiovascular disease; HF, heart failure; ACS, acute coronary syndromes; CVA, cerebrovascular accidents. There were a total of 6,529 distinct admissions. The HF admissions category included HF only, HF & ACS, and HF & CVA. The ACS admissions category included ACS only and ACS & CVA. At the time of the analysis (September 5, 2020), a total of 47 patients remained hospitalized and were excluded from length of stay and in-hospital mortality calculations (26 HF, 7 ACS, 14 CVA).

† Values are mean ± SD for continuous variables or n (%) for categorical variables

| **Supplemental Table 4: Exploratory Analyses of Acute Heart Failure Admission Characteristics and Illness Severity across the Duke Health System from March 2 to August 31 Separated by the North Carolina Stay-At-Home Order with 2019 as Reference Groups*** | | | | | | | |
| --- | --- | --- | --- | --- | --- | --- | --- |
| **HF Admission Groups, N** | | **Mar 2 – Mar 29, 2019**  **(285)** | **Mar 2 – Mar 29, 2020**  **(203)** | **p^†^** | **Mar 30 – Aug 31, 2019**  **(1,514)** | **Mar 30 – Aug 31, 2020**  **(1,186)** | **p^‡^** |
| **TTE**^§^ | **LVEF %, mean (SD)** | 37.6 (14.8) | 41.3 (14.6) | ***0.030*** | 38.4 (15.2) | 37.2 (15.4) | 0.075 |
|  | **LV Mass in g grams, mean (SD)** | 254.9 (102.2) | 267.2 (110.8) | 0.32 | 273.2 (104.2) | 276.6 (104.9) | 0.51 |
| **ADHERE** | **Mortality Risk Group, N (%)** |  |  |  |  |  |  |
|  | **Low** | 146 (51.2) | 114 (56.1) | 0.40 | 779 (51.5) | 588 (49.6) | ***0.018*** |
|  | **Intermediate** | 129 (45.3) | 85 (41.9) |  | 697 (46.0) | 545 (45.9) |  |
|  | **High** | 10 (3.5) | 4 (2.0) |  | 38 (2.5) | 53 (4.5) |  |
| **GWTG-HF** | **Mortality Risk Group, N (%)** |  |  |  |  |  |  |
|  | **<1%** | 51 (17.9) | 51 (25.1) | 0.087 | 324 (21.4) | 248 (20.9) | ***0.037*** |
|  | **>1-5%** | 197 (69.1) | 118 (58.1) |  | 1,022 (67.5) | 762 (64.3) |  |
|  | **>5-10%** | 29 (10.2) | 25 (12.3) |  | 117 (7.7) | 121 (10.2) |  |
|  | **>10%** | 8 (2.8) | 9 (4.4) |  | 51 (3.4) | 55 (4.6) |  |

* HF, heart failure; LOS, length of stay; TTE, transthoracic echocardiography; LVEF, left ventricular ejection fraction; LV, left ventricular; ADHERE, “Acute Decompensated Heart Failure National Registry Algorithm”; GWTG-HF, Get with The Guidelines® - Heart Failure Risk Score. The HF admissions category included HF only, HF & ACS, and HF & CVA. Groups within 2019 and 2020 were divided into before or after March 30 since the North Carolina Stay at Home order went into effect on March 30, 2020 and following the first North Carolina COVID-19 case.

† P-value compares difference between heart failure admission groups in March 2 – March 29, 2019 vs. 2020.

‡ P-value compares difference between heart failure admission groups in March 30 – August 31, 2019 vs. 2020.

§ There were 2,129 available TTE studies of the 3,188 total HF admissions during the above periods. Comparisons for LVEF are of 2,129 TTEs (189 between Mar 2 – Mar 29, 2019; 1,017 between Mar 30 – Aug 31, 2019; 127 between Mar 2 – Mar 29, 2020; 796 between Mar 30 – Aug 31, 2020). Comparisons for LV mass are of 2,038 TTEs (181 between Mar 2 – Mar 29, 2019; 964 between Mar 30 – Aug 31, 2019; 122 between Mar 2 – Mar 29, 2020; 771 between Mar 30 – Aug 31, 2020).

**Pairwise comparisons for significant differences in Tables 2 and 3**

**Reference comparisons: #1) Jan 1-Mar 29, 2019; #2) Mar 30-Aug 31, 2019; #3) Jan 1-Mar 29, 2020; #4) Mar 30-Aug 31, 2020**

- **For mean acute CVD daily admissions (Table 2):**

**1 vs 2: -0.27, p = 0.98; 1 vs 3: -1.67, p=0.13; 1 vs 4: -3.72, p<0.001 ; 2 vs 3: -1.41, p=0.17 ; 2 vs 4: -3.45, p<0.001; 3 vs 4: -2.04, p=0.016**

- **For mean acute CVD length of stay (Table 2):**

**1 vs 2: -0.61, p= 0.20; 1 vs 3: -0.14, p= 0.98; 1 vs 4: -0.96, p= 0.016; 2 vs 3: +0.47, p= 0.46 ; 2 vs 4: -0.35, p= 0.59; 3 vs 4: -0.82, p= 0.064**

- **For mean acute HF daily admissions (Table 2):**

**1 vs 2: -0.22, p=0.96; 1 vs 3: -0.94, p=0.23; 1 vs 4: -2.34, p<0.001; 2 vs 3: -0.72, p=0.36; 2 vs 4: -2.12, p<0.001; 3 vs 4: -1.39, p=0.009**

- **For mean acute HF length of stay (Table 2):**

**1 vs 2: -1.18, p= 0.058; 1 vs 3: -0.71, p= 0.55; 1 vs 4: -1.61, p= 0.006; 2 vs 3: +0.47, p= 0.77 ; 2 vs 4: -0.44, p= 0.74; 3 vs 4: -0.90, p= 0.28**

- **For mean acute ACS daily admissions (Table 2):**

**1 vs 2: +0.08, p=0.99; 1 vs 3: -0.35, p=0.71; 1 vs 4: -0.75, p=0.057; 2 vs 3: -0.43, p=0.46; 2 vs 4: -0.83, p=0.006; 3 vs 4: -0.39, p=0.54**

- **For GWTG-HF >10% Risk Group among HF admissions (Table 3):**

**1 vs 2: -0.5%, p=0.38; 1 vs 3: +1.47%, p=0.087; 1 vs 4: +0.77%, p=0.61; 2 vs 3: +1.97%, p=0.010; 2 vs 4: +1.27%, p=0.037; 3 vs 4: -0.7%, p=0.45**
